# Regulation of histone H3K27 methylation in inflammation and cancer

**DOI:** 10.1186/s43556-025-00254-x

**Published:** 2025-03-05

**Authors:** Jing Ma, Yalin Zhang, Jingyuan Li, Yanqi Dang, Dan Hu

**Affiliations:** 1 Seventh People’s Hospital of Shanghai University of Traditional Chinese Medicine, No. 358 Datong Road, Pudong New Area, Shanghai, 200137 China; 2 Institute of Digestive Diseases, Longhua Hospital, China-Canada Center of Research for Digestive Diseases (ccCRDD), Shanghai University of Traditional Chinese Medicine, Shanghai, 200032 China; 3 State Key Laboratory of Integration and Innovation of Classic Formula and Modern Chinese Medicine, (Shanghai University of Traditional Chinese Medicine), Shanghai, 200032 China

**Keywords:** Inflammation, Cancer, Epigenetic regulation, H3K27 methylation

## Abstract

Inflammation is a multifaceted defense mechanism of the immune system against infection. Chronic inflammation is intricately linked to all stages of tumorigenesis and is therefore associated with an elevated risk of developing serious cancers. Epigenetic mechanisms have the capacity to trigger inflammation as well as facilitate tumor development and transformation within an inflammatory context. They achieve this by dynamically modulating the expression of both pro—inflammatory and anti—inflammatory cytokines, which in turn sustains chronic inflammation. The aberrant epigenetic landscape reconfigures the transcriptional programs of inflammatory and oncogenic genes. This reconfiguration is pivotal in dictating the biological functions of both tumor cells and immune cells. Aberrant histone H3 lysine 27 site (H3K27) methylation has been shown to be involved in biological behaviors such as inflammation development, tumor progression, and immune response. The establishment and maintenance of this repressive epigenetic mark is dependent on the involvement of the responsible histone modifying enzymes enhancer of zeste homologue 2 (EZH2), jumonji domain containing 3 (JMJD3) and ubiquitously transcribed tetratricopeptide repeat gene X (UTX) as well as multiple cofactors. In addition, specific pharmacological agents have been shown to modulate H3K27 methylation levels, thereby modulating inflammation and carcinogenesis. This review comprehensively summarises the current characteristics and clinical significance of epigenetic regulation of H3K27 methylation in the context of inflammatory response and tumor progression.

## Introduction

Epigenetic control refers to heritable modifications in gene activity that do not change the DNA structure. This involves the suppression of certain genes through epigenetic mechanisms, leading to transcriptional inhibition during the process of growth and cellular specialization. Active or silent gene states are controlled by the addition or removal of epigenetic modifications in the chromatin. Chromatin is made up of nucleosomes, which are composed of 147 DNA base pairs coiled around core histones (H2A, H2B, H3, and H4).

Histone lysine methylation, one of these chromatin modifications, plays a fundamental role in regulating chromatin structure. Lysine residues can be mono-, di-, or trimethylated, resulting in various combinations that establish specialized chromatin states. Commonly, genes that are actively being transcribed show peaks of trimethylation of histone H3 lysine 4 (H3K4me3) near their promoters, whereas inactive genes are characterized by extensive regions of dimethylation of histone H3 lysine 9 (H3K9me2), H3K9me3, or trimethylation of histone H3 lysine 27 (H3K27me3) [1]. Epigenetic regulation, along with genetic abnormalities, especially in chromatin, is crucial for regulating inflammatory genes and driving chronic inflammatory diseases and cancer.

**Fig. 1:**
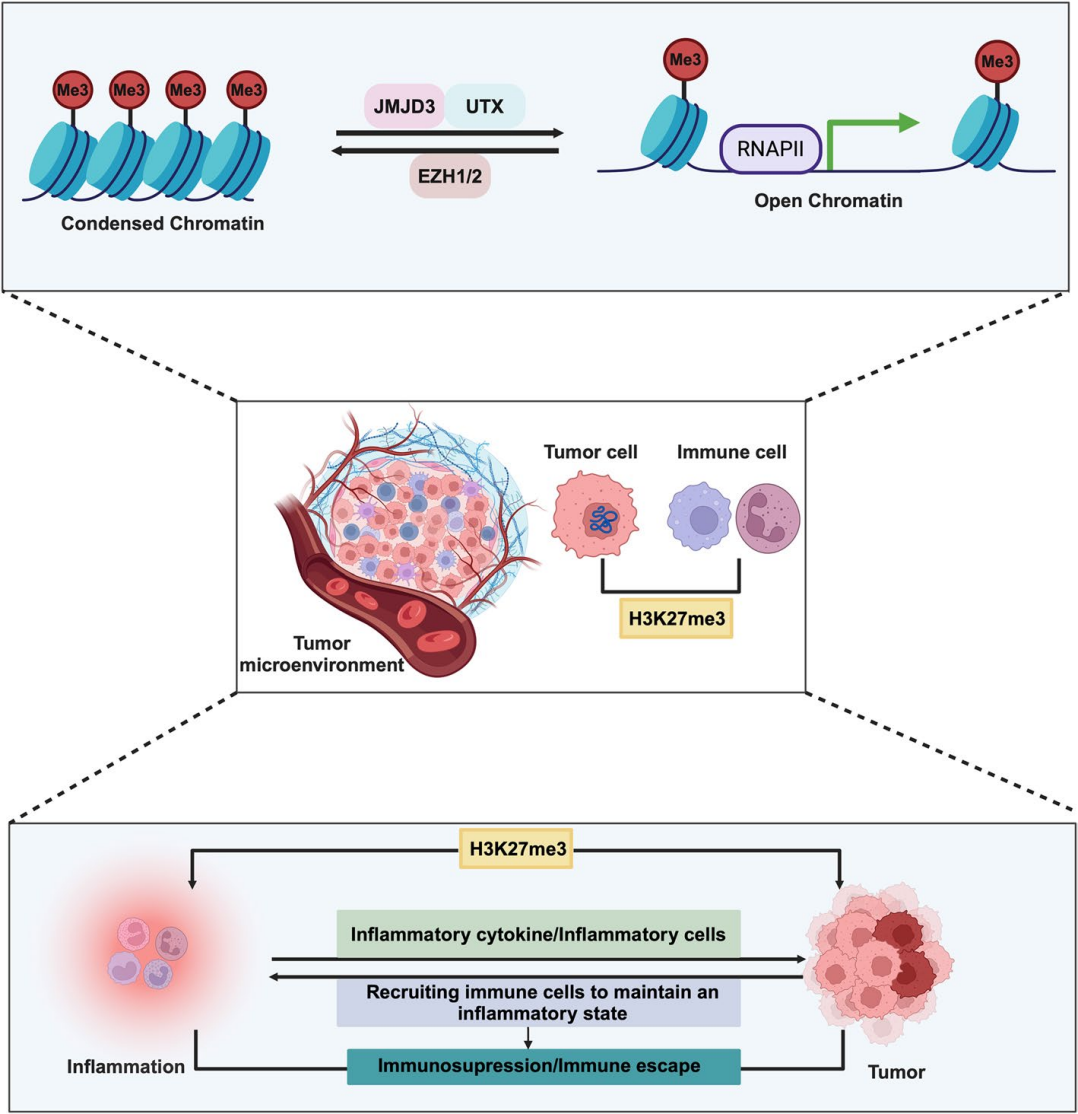
Role of H3K27 methylation modification in inflammation and cancer. H3K27 methylation modification is a repressive epigenetic modification whose level of regulation is dependent on histone methylation transferase EZH2 and histone demethylation transferase JMJD3 and UTX. Studies have demonstrated that H3K27me3 plays an important role in inflammation and carcinogenesis. In addition to this, immune cells in the tumor microenvironment participate in the inflammatory response, and secretion of immune cells of inflammatory cytokines and recruitment of immune cells help tumor cells to evade immune surveillance, and the suppressive tumor immune response formed by this process is a key factor in inducing tumorigenesis. Figure was created in https://BioRender.com

Inflammation is the innate defense mechanism against harmful stimuli such as pathogens, damaged cells or irritants. This complex immune response aims to neutralize threats and restore tissue structure and function to a baseline state [2, 3]. In recent years, the intricate relationship between inflammation and tumors has been continuously expanding in scope and being further elucidated [4, 5]. Within this context, epigenetic modifications have emerged as pivotal mechanisms underpinning the progression of both inflammation and tumors. Chronic inflammation is widely regarded as a key determinant in triggering tumorigenesis [6]. The dynamic modulation of chromosomal conformation exerts an impact on the secretion of relevant inflammatory factors. These factors, in turn, initiate inflammation—associated carcinogenic cascades, ultimately leading to the malignant transformation of tumor cells. Inflammatory cytokines influence immune responses and the tumor microenvironment, where inflammation can both enhance immune surveillance and, under certain conditions, suppress tumor-specific immune responses. This creates a favorable environment for tumor cells to evade immunosurveillance, promoting tumorigenesis and progression [7]. Genes and signalling pathways implicated in tumor progression not only support the sustenance of the biological characteristics of tumors but also prompt a succession of immune cells to infiltrate the tumor microenvironment, thereby preserving an inflammatory milieu. Under the influence of epigenetic modifications, aberrant restrictive or permissive responses emerge as pivotal determinants fueling the advancement of both inflammation and cancer. H3K27 methylation has been implicated in regulating inflammatory gene transcription and is involved in the pathogenesis of chronic inflammation-associated diseases [8]. Furthermore, maintaining H3K27 methylation is critical for tumor development, as epigenetic modifiers that maintain this mark can directly regulate oncogenes and tumor suppressors, influencing tumor behavior. However, enzymes such as enhancer of zeste homologue 2 (EZH2), jumonji domain containing 3 (JMJD3) and ubiquitously tanscribed tetratricopeptide repeat gene X (UTX) exhibit dual roles in cancer, where their effects on tumorigenesis can be dependent on or independent of their methyltransferase activity [9] (Fig. 1).

Drugs targeting H3K27 methylation, known as epimedicines, are currently being evaluated in preclinical and clinical settings for their potential in treating inflammation and cancer. The EZH2 inhibitor Tazemetostat has been approved for treating epithelioid sarcoma [10], and other small-molecule inhibitors have shown enhanced anticancer efficacy when combined with other therapies. However, many epimedicines still face challenges such as toxicity, drug resistance, and short half-lives, making their development a complex endeavor [11].

This review presents the literature on H3K27 methylation in inflammation and tumor progression, highlighting the complex regulatory roles of associated epigenetic modifiers and their potential molecular mechanisms in disease. It broadens the "tumor cell-centric" perspective to include the tumor microenvironment, exploring aberrant genetic and epigenetic alterations. Additionally, the review discusses the therapeutic potential and prospects of drugs targeting H3K27 methylation in these biological processes.

## Biological basis of H3K27 methylation

### H3K27 types of methylation

The methylation of H3K27 in mouse embryonic stem cells, analyzed by tandem mass spectrometry, revealed that the predominant methylation mark was H3K27me2, followed by H3K27me3 and H3K27me1. These three methylation marks, despite their functional exclusivity and distinct genome-wide distributions, are closely linked in regulating chromatin status and gene expression [12]. H3K27me3, the most studied and prominent methylation modification, is primarily found at gene promoters and enhancers, where it represses transcription and plays a role in cell differentiation and tumor progression. H3K27me2, the most widely distributed form of H3K27 methylation in mammals and Drosophila, is concentrated in regions of low transcriptional activity. It is thought to suppress aberrant enhancer activation and prevent conversion to H3K27me3 [13].

While the biological significance of H3K27me2 is less understood, it is known to be polycomb repressive complex 2 (PRC2)-dependent, ubiquitously distributed throughout the genome, and considered the default state for H3K27. H3K27me2 is found in most euchromatin regions in mammals and Drosophila, excluding H3K27me1, H3K27ac, and H3K27me3. The enzymatic process of H3K27me2 is more straightforward for PRC2, requiring only transient interactions, unlike H3K27me3, which needs more stable associations and accumulates slowly [14, 15]. Deletion of PRC2 in Drosophila cells results in higher expression of intragenic mRNAs and raised levels of H3K27ac at locations previously tagged with H3K27me2 [16]. H3K27me1, found exclusively at actively transcribed genes, stimulates transcription through PRC2 activity and is regulated by H3K36me3 [12]. H3K27 methylation regulates transcription on a genome-wide scale through distinct molecular mechanisms, highlighting the specificity of methyltransferases in substrate selection and the complexity of the PRC2 complex in epigenetic regulation.

### H3K27 methylation-related enzymes

**Fig. 2:**
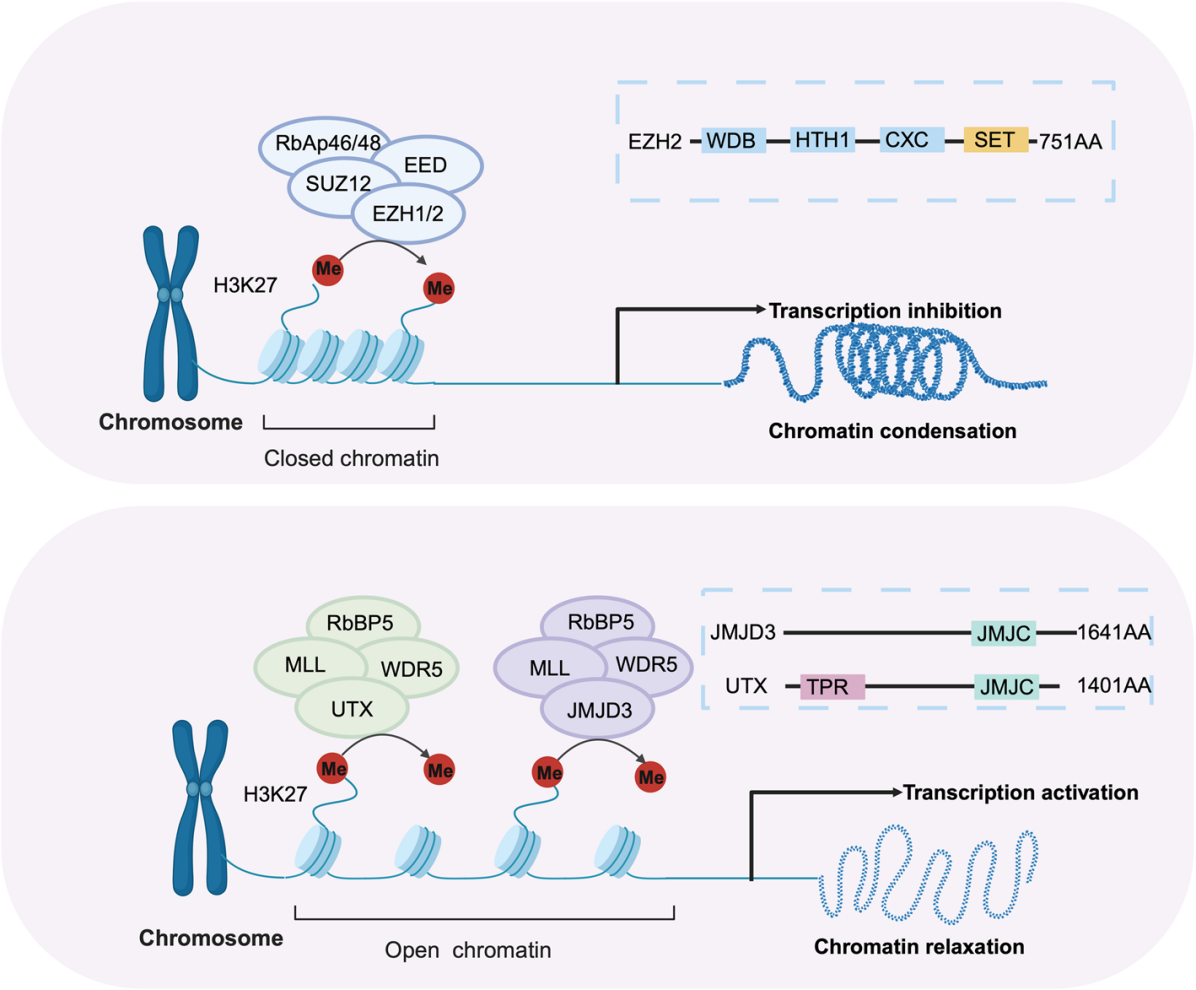
Regulation of H3K27 methylation epigenetic related enzymes. Establishment and maintenance of H3K27 methylation is dependent on the combined action of the histone methyltransferase EZH2 and the histone demethyltransferases JMJD3 and UTX. EZH2 is a component of the PRC2 multiprotein chromatin modification complex with four structural domains the WD-binding structural domain, PRC2 HTH 1 structural domain, the CXC structural domain, and the SET structural domain, where the SET structural domain is responsible for methyl installation at the histone H3 lysine 27 site. EZH2 regulates chromatin compaction and mediates transcriptional repression. JMJD3 and UTX are histone demethyltransferases responsible for removing the methyl group installed at the histone H3 lysine 27 site. Both have the JMJC structural domain, with the exception of UTX, which has six TPR domains at the N-terminus. JMJD3 and UTX are able to maintain chromatin relaxation and activate transcription of specific genes. Figure was created in https://BioRender.com

The establishment and maintenance of H3K27 methylation states are regulated by methyltransferases and demethylases, along with their cofactors. These modifications influence chromatin dynamics, cell behavior, and are strongly implicated in inflammation and cancer progression (Fig. 2).

#### Histone methyltransferases

The PRC2 protein complex, with its catalytic subunit EZH2, is responsible for adding methyl groups to lysine 27 of histone H3. EZH2 uses S-adenosylmethionine (SAM) to catalyze dimethylation and trimethylation at H3K27, with the cooperation of other PRC2 components and cofactors [17]. The SET domain containing at the C-terminal end of EZH2 catalyzes the methylation, and its activation is dependent on the VEFS domains of the embryonic ectoderm development (EED) and SUZ12 polycomb repressive complex 2 subunit. Notably, H3K27me3 interacts with EED, promoting a positive feedback loop that enhances PRC2 activity and supports its epigenetic silencing function [18].

Mutations or overexpression of EZH2 are linked to various cancers, as EZH2-mediated changes in H3K27me3 lead to chromatin compaction and transcriptional silencing, contributing to inflammation and tumor progression [19]. Overexpression of EZH2 and H3K27me3 levels has been shown to induce peritoneal metastasis in triple-negative breast cancer models [8, 20]. Interestingly, recent studies have revealed that EZH2 can also influence genetic information transmission independent of PRC2-mediated transcriptional repression. In these studies, H3K27me3 activated transcription of the KPT14 gene by inhibiting the transcription factor SP1, enhancing metastatic potential in triple-negative breast cancer [20]. In acute leukemia, EZH2 utilizes a hidden transactivation domain in conjunction with cMyc and p300 to activate gene expression, contributing to tumorigenesis alongside its classical role in transcriptional repression [21].

#### Histone demethylases

To maintain active differentiation status and DNA damage repair function, tumor cell plasticity requires epigenetic regulators involved in histone demethylation modifications to balance the H3K27 methylation deposited by the PRC2 complex. The two key demethylases, JMJD3 and UTX, belong to the evolutionarily conserved Jumonji C (JmjC) family, each possessing a JmjC structural domain [22]. These demethylases convert H3K27me2 and H3K27me3 to the monomethylated H3K27 state, and enrichment of H3K27me1 at transcriptionally active genes counters EZH2-mediated transcriptional repression, thereby influencing cell fate [23]. The UTX gene, located on the X chromosome, is widely expressed and contains six tetratricopeptide repeat domains at the N-terminus and a JmjC domain at the C-terminus, which together exert demethyltransferase activity, removing H3K27me2 and H3K27me3 without affecting other methylated residues [22]. The Y-chromosome homologue, UTY, has limited methyltransferase activity, likely due to structural changes in the JmjC domain [24]. JMJD3, a gene like UTX, is another demethylase that plays a role in epigenetic reprogramming.

JMJD3 has a unique advantage in chromatin remodeling that does not rely on H3K27 demethylase activity. It induces interactions between the T-box transcription factor and the SWItch/sucrose non-Fermentable complex in differentiated cells, facilitating gene transcription initiation [25]. JMJD3 also acts as a downstream target of the interleukin-4 (IL-4)/Janus kinase 3 (JAK3)/signal transducer and activator of transcription (STAT) 6 axis, contributing to macrophage M2 polarization through histone demethylation and activation of IRF4 transcription. The JMJD3-interferon regulatory factor 4 (IRF4) axis has been implicated in renal fibrosis progression [26]. Additionally, metabolic signaling affects JMJD3-IRF4-mediated macrophage polarization. Specifically, reducing the α-ketoglutarate/succinate ratio inhibits this polarization and alleviates inflammatory pain symptoms [27]. Furthermore, upregulation of JMJD3 by nuclear factor kappa-light-chain-enhancer of activated B cells (NF-κB) contributes to inflammatory diseases, with knockdown of JMJD3 in human leukemia monocytes suppressing chemokine and NF-κB-related inflammatory gene expression [28].

## H3K27 methylation in inflammation

Inflammation constitutes the innate defense of body mechanism against deleterious stimuli, such as pathogens, damaged cells, or irritants. This complex immune response is orchestrated to neutralize threats and restore tissue architecture and functionality to their baseline state [2, 3]. Clinically, inflammation is characterized by erythema, edema, pain, and sometimes impaired function, with its regulation predominantly mediated by inflammatory cytokines [29–31]. Proinflammatory cytokines amplify the inflammatory response, whereas anti-inflammatory cytokines attenuate it.

In acute inflammation, the reaction of body to a trigger includes a higher movement of white blood cells and fluid from the bloodstream to the affected regions. Inflammation that persists for an extended period of time is called chronic inflammation. Symptoms of chronic inflammation are slightly less severe than acute inflammation symptoms, but they are persistent [32, 33]. Persistent inflammation has been linked to a range of health conditions, such as cancer [5, 34], diabetes [35–37], cardiovascular disorders [38], neurological diseases [39, 40], rheumatoid arthritis [41, 42], systemic lupus erythematosus [43, 44], and asthma [45]. It is a significant risk factor for major types of cancer, contributing to approximately 25% of cases. Persistent inflammation is associated with different phases of cancer development, such as cell changes, tumor advancement, growth, spread, infiltration, blood vessel formation, and spread to other parts of the body [46, 47].

Signal-specific and gene-specific functions are managed by a complex regulatory network during the inflammatory response. The macrophage signaling network activates genes responsible for antimicrobial defense, immune response, and tissue remodeling to contribute the development of chronic inflammatory diseases [48, 49]. Data analysis indicates that epigenetic regulation is crucial for the development of the macrophage phenotype [50, 51].

Hypermethylation of H3K27, catalyzed by EZH2, is associated with condensed chromatin and gene silencing in inflammatory responses. Silenced genes with anti-inflammatory functions, as well as inflammatory cytokines are affected by this modification. EZH2 enhances T-cell viability by methylating Vav, which is essential for actin formation in T-cell receptor activation [52]. Research indicated that the suppression of IL4 and Interleukin-13 genes in T helper (Th)1 cells is associated with EZH2-induced H3K27me3 [53, 54]. To reduce liver failure in mice, EZH2 could enhance H3K27me3 enrichment, thereby activating the signaling pathways of NF-κB and protein kinase B (AKT) [55]. H3K4me3 and H3K27me3 levels in the promoters of STAT1, STAT3, and retinoic acid receptor-related orphan receptor gamma t play a crucial role in determining the fate of Th17 cells via interferon gamma signaling [56, 57]. Dendritic (DC) cells derived from monocytes are also influenced by H3K4me3 and H3K27me3 modifications in tumors and inflammatory environments [58, 59]. When there is severe systemic inflammation, NF-κB/RelB-dependent silencing represses genes [60]. Endotoxin activation is required and adequate for suppressing acute proinflammatory genes through RelB induction [61]. Infusion of DC cells alleviated colitis in mice with inflammatory bowel disease by inhibiting lymphocyte proliferation, associated with high H3K27me3 and low H3K4me3 levels at interferon regulatory factor 8 promoters [62]. Moreover, EZH2 plays a role in controlling chromatin organization at the transgelin promoter by influencing H3K27me3 levels, serving as a mediator of interleukin 1 beta (IL-1β) and transforming growth factor beta (TGF-β) 2 signaling pathways [63, 64]. Chronic inflammation is mediated by proinflammatory M1 macrophages in the process of wound healing, especially in individuals with type 2 diabetes. The process of bone marrow stem and progenitor cells transforming into macrophages includes an increase of H3K27me3 at the IL-12 gene promoter, leading to the suppression of IL-12 expression [65]. Histone methylation at H3K27 is also associated with inflammation and healing processes in dental pulp. Inflammation reduces H3K27me3 in pulp tissue and cells, potentially inducing dental pulp regeneration [66, 67]. Involvement of EZH2 in these mechanisms suggests it could serve as an epigenetic marker and regulator of dental pulp inflammation, with potential applications in dental pulp regeneration. Exposure to specific environments, such as colitis, can induce aberrant H3K27me3, implicating abnormal histone modification in the formation of defects [68, 69].

Histone demethylation occurs when histone hypermethylation is converted to hypomethylation. Enzymes with a Jumonji C domain catalyze this reaction by removing mono-, di-, and trimethyl groups. JMJD3, a member of the Jumonji domain-containing protein family, is also referred to as lysine-specific demethylase 6B. This family also consists of UTX. JMJD3 decreases the levels of H3K27me2 and H3K27me3 [70, 71]. Inactive gene promoters are usually linked with trimethylated H3K27, whereas active gene promoters are linked with monomethylated H3K27 [72, 73]. Several studies have reported that JMJD3 and UTX can activate genes involved in the inflammatory response. When bacterial components and inflammatory cytokines are exposed to macrophages, JMJD3 promotes both inflammatory and anti-inflammatory reactions, which is crucial for the prevention of infections and the healing of wounds [50, 74]. H3K27me3 levels and transcriptional function are controlled by attaching to polycomb group (PcG) target genes. JMJD3 erases histone marks that control differentiation and cell identity, linking inflammation to epigenome reprogramming and macrophage plasticity, which may explain differentiation abnormalities in chronic inflammation. When IL-4 is administered continuously, JMJD3 is activated and the STAT6 is repressed by H3K27me3 [50, 75]. Binding of activated STAT6 to the promoter of JMJD3 leads to its positive regulation, which in turn triggers the expression of specific inflammatory genes by removing H3K27 methylation [76]. JMJD3 can also function independently of H3K27 demethylation, being specifically attracted to transcription start sites marked by H3K4me3 and the RNA Polymerase II complex. This indicates that the mutual exchange of H3K4 and H3K27 methylation plays a crucial role in epigenetic regulation of genes. Several genes that are critical to mammalian development and differentiation are targeted by PcGs in the genome. Polycomb proteins bind to control regions of specific genes and promote DNA methyltransferase activity, effectively suppressing their expression [77]. Knockdown of JMJD3 in THP-1 cells reduces inflammatory markers and heat shock protein beta-1, but increases glutathione peroxidase and glia maturation factor-gamma expression [78]. As a consequence of the modifications, they affect the NF-κB, chemokine, and CD40 pathways, and blocking JMJD3 suppresses multiple NF-κB-regulated inflammatory genes [78, 79]. Bacterial toxin lipopolysaccharide (LPS) activates NF-κB signaling to reduce JMJD3 levels in keratinocytes derived from human gingival tissue, indicating that endogenous JMJD3 could potentially regulate the expression of proinflammatory cytokines [78, 80]. JMJD3 controls the expression of macrophages and plays a crucial role in foam cell formation, indicating a potential therapeutic function in atherosclerosis [75, 81]. Notably, JMJD3 expression is upregulated in macrophages in diabetic wounds, a process that relies on its positive regulation by the cytokine IL-6 and mediates inflammation through the JAK1,3/STAT3 signaling pathway [82]. H3K27me3 regulates mycobacterial infection, which contributes to foamy macrophage generation. In diabetics, an increase in H3K27me3 suppresses the expression of IL-12, which is produced by macrophages in response to JMJD3, and inhibiting JMJD3 prevents this process [83]. This suggests that inhibiting JMJD3 could treat diabetic wounds. Higher levels of M1-type macrophages were observed in tissues with periodontitis induced by type 2 diabetes mellitus. Additionally, reduced expression of JMJD3 in monocytes plays a key regulatory role in controlling M2-type macrophage polarization [84]. Another study focused on diabetes-induced inflammation of the male reproductive system, suggesting that hyperglycemia-driven M1-type macrophage polarization in the testes may contribute to male infertility. Notably, the induction of M2-type macrophages by JMJD3 was found to effectively intervene in the progression of infertility [85]. NF-κB-JMJD3 interactions enhance gene activation for inflammatory, matrix metalloproteinases, and growth factors through demethylation of H3K27me3 [74, 86, 87]. It is found that keratinocytes fail to heal correctly, when JMJD3 or NF-κB is disabled. JMJD3 acts as a bridging molecule in transcription initiation, a function independent of its demethylase activity. It is associated with stanniocalcin1 and chemokine (C–C motif) ligand 2 in human malignant melanomas [86, 88]. JMJD3 plays a crucial role in promoting M2 microglia polarization; blocking JMJD3 hinders the shift to M2 phenotype and enhances M1 microglial inflammatory reactions [89]. The control of JMJD3 gene expression by STAT1 and STAT3 is essential for initiating inflammatory reactions in microglia, regardless of its demethylase function [90]. Moreover, JMJD3 could reduce H3K27me3 level of hyaluronan synthase 2 to regulate muscle stem cells quiescence, finally to drive muscle regeneration [91]. By promoting H3K27-H3K4 demethylation, JMJD3 inhibits levels of IL4i1 or adenosine A2a and promotes acute lung injury by increasing the macrophage polarization [92, 93]. JMJD3 has a crucial function in regulating non-infectious inflammation, such as in atherosclerosis and the healing of wounds. Targeting JMJD3 may have clinical benefits depending on the disease state.

UTX mediates the demethylation of H3K27me3 at the arachidonate 15-lipoxygenase (ALOX15) promoter following IL-4 treatment, facilitating ALOX15 transcriptional activation [94, 95]. This IL-4-induced H3K27me3 demethylation and subsequent ALOX15 induction were significantly reduced when UTX expression was knocked down. UTX enhances chromatin activity by increasing H3K4 methylation and decreasing H3K27 methylation at specific regions of the HIV-1 long terminal repeat [96, 97]. Enhancing the movement of NF-κB p65 to the nucleus and its interaction with HIV-1 long terminal repeat increases the expression of HIV-1 genes. The demethylase activity of H3K27 is crucial for the UTX induced increase in HIV-1 transactivation [96] (Table 1).

**Table 1:** The role of histone modifications including H3K27 methylation in the progression of inflammation

Histone Specificity	Inflammatory effects regulated by histone modifications	Mediated Signaling	Mediated Genes	Refs
H3K27me3	EZH2-mediated H3K27me3 induces liver failure by triggering pro-inflammatory cytokines	NF-κB and Akt signalling pathways	Tumor necrosis factor (TNF) and TGF-β1	[55]
	JMJD3 activates IL12 expression via H3K27me3 and promotes the reprogramming of the pro-inflammatory phenotype of macrophages and chronic inflammation in type 2 diabetes wounds	-	IL12	[65]
	EZH2-mediated hypomethylation plays an anti-inflammatory role and enhances the differentiation ability of human dental pulp cells	-	TNF-a, IL-1β, IL-6 and IL-8	[66]
	EZH2 deficiency promotes inflammatory response and apoptosis in colitis	NF-κB signalling pathways	TNFα and E3 ubiquitin—protein ligase ITCH	[98]
	Serum amyloid A induces JMJD3 expression, which promotes the formation of foam macrophages, neutrophil accumulation, and the transcription of multiple inflammatory cytokine genes	NF-κB signalling pathways	Interleukin—23 p19 subunit, Granulocyte colony—stimulating factor and Triggering receptor expressed on myeloid cells 1	[81]
	JMJD3 participates in normal wound repair by inducing the transcription of inflammatory genes and macrophage-mediated inflammation through the H3K27me3 mechanism	JAK1,3/STAT3 and NF-κB signalling pathways	Stimulator of interferon genes	[82]
	JMJD3 promotes the invasive growth of melanoma by mediating macrophage infiltration through the H3K27me3 mechanism	NF-κB, Bone morphogenetic protein and PI3K signalling pathways	Stanniocalcin 1, CCL2 and Bone morphogenetic protein 4	[99]
	JMJD3 promotes muscle stem cell adaptation to inflammation and enhances muscle repair through the H3K27me3 mechanism	-	Hyaluronan synthase 2	[91]
H3K4me3	JMJD3 exacerbates lipopolysaccharide-induced acute lung injury through the H3K27me3-H3Kme3 mechanism	-	IL- 4-induced gene-1	[92]
	H3K4me3 and K3K27me3 drive the differentiation of monocyte-derived DCs in the tumor and inflammatory environment	TGF-β signalling pathways	NFκB2, STAT5A, STAT5B and Nuclear factor of activated T—cells	[58]
H3K4me2	UTX promotes the expression of IL-6 through the H3K27me3 mechanism and can also mediate the involvement of H3K4me2 in the inflammatory process during enhancer activation	-	Mixed—lineage leukemia 4 and Interferon—β	[100]
H3K27ac	IL-4 stimulation upregulates H3K9ac and H3K27ac expression and downregulates H3K9me3 and H3K27me3 expression, which is involved in the development of asthma	IL-4/STAT6 signalling pathways	Igε	[101]
	IL-1-dependent upregulation of H3K27ac is involved in sterile cytokine inflammation	NF-κB signalling pathways	Transforming growth factor—β—activated kinase 1, IκB kinase 2 and p65	[102]

Histone acetylation is another form of histone modification that contrasts with histone methylation. Two sets of enzymes, histone acetyltransferases (HATs) and histone deacetylases, control this procedure. HATs activate inflammatory genes through histone acetylation, whereas increased histone deacetylases activity represses these genes. The HAT acetylates promoters of multiple proinflammatory cytokines (IL-1, IL-2, IL-8, and IL-12), resulting in their activation at the transcriptional level [103, 104]. Silencing of p300, a widely recognized HATs, has been demonstrated to impede the growth and ability of dental pulp cells to differentiate into tooth tissues, indicating the important involvement of HAT in inflammatory reactions within the pulp [67, 105]. IL-4 treatment increases HAT activity in Ramos cells, leading to higher expression of H3K9ac and H3K27ac, and decreases of H3K9me3 and H3K27me3 [106, 107]. Furthermore, IL-4 boosts H3 acetylation in the IgE promoter areas [108]. In endometriosis, targeting prostaglandin E2 receptors EP2 and EP4 selectively can alter multiple pathways, leading to a reduction in H3K27me3 levels and an increase in H3K27ac expression [109, 110]. Hemodynamic shear forces, which help prevent valve calcification, trigger anti-bone formation and anti-inflammatory pathways via notch receptor 1 (NOTCH1), leading to changes in H3K27ac levels at NOTCH1-linked enhancers and influencing the expression of genes related to bone formation, inflammation, and oxidative stress [111, 112]. IL-1 triggers H3K27ac and p65 attachment, and blocking TGF-beta activated kinase 1 or inhibitor of nuclear factor kappa B kinase subunit beta, as well as removing p65, prevents enhancer activation and gene expression induced by IL-1 [102, 113, 114].

## H3K27 methylation in cancer

### Role of H3K27 methylation in tumor progression

Epigenetic modifications play a critical role in the transformation of quiescent cells into malignant cells. Several cancer genome sequencing studies have shown that H3K27 methylation levels and associated epigenetic regulators fluctuate at the gene level. These modifications contribute to tumor cell survival and adaptation by regulating the transcriptional activity of oncogenes, thereby influencing cancer cell differentiation and plasticity [115–117] (Table 2).

**Table 2:** Role of H3K27 methylation-related epigenetic modifying enzymes in tumors

Modifying enzyme	Roles in cancer	Cancer type	Fuction	Ref
EZH2	Pro-cancer	Breast cancer	High-level expression of EZH2 promotes epithelial mesenchymal transition during tumor cell proliferation and lymph node metastasis	[118]
		Ovarian cancer	High-level expression of EZH2 blocks the cell cycle and promotes cell proliferation in vitro after transfection of ovarian cancer stem cells with miRNA-98	[119]
		Colorectal cancer	Solute carrier family 34 member 2 upregulates EZH2 expression to promote proliferation and chemoresistance to apoptosis in colorectal cancer	[120]
		Triple-negative breast cancer	MEK-ERK1/2-Elk-1 pathway upregulates EZH2 expression and promotes tumor cell proliferation, invasion and poor prognosis	[121]
	Anti-cancer	Lung adenocarcinoma	Activation of downstream pathways Akt and ERK in Ezh2 deletion promotes tumor transformation	[122]
		Pancreatic cancer	EZH2 deficiency inhibits pancreatic tissue repair and accelerates KRas(G12D)-driven neoplasia	[123]
		Myelodysplastic syndrome	EZH2 point mutations lead to lower overall survival rates	[124]
		T-ALL	EZH2 silencing increases the in vivo tumor-forming potential of human T-ALL cells transplanted into immunodeficient mice	[125]
UTX	Anti-cancer	Bladder cancer	UTX mutation down-regulates insulin-like Growth Factor Binding Protein 3 expression thereby inducing bladder cancer tumorigenesis and high recurrence rate	[126]
		Pancreatic cancer	Down-regulation of UTX expression inhibited epithelial mesenchymal transition and typical differentiation mediated by GATA Binding Protein 6 and effectively suppressed malignant progression to pancreatic cancer	[127]
		Kidney cancer	Downregulation of UTX expression induces poor prognosis in clear cell renal cell carcinoma	[128]
	Pro-cancer	Breast cancer	Synergistic Regulation of UTX and Mixed-Lineage Leukemia 4 Promotes Breast Cancer Cell Proliferation, Invasion, and Poor Prognosis	[129]
			UTX and C-X-C chemokine receptor type 4 together promote metastasis and progression of estrogen receptor-positive breast cancer	[130]
		T-ALL	UTX and T-cell acute lymphocytic leukemia 1 together maintain T-ALL progression	[131]
JMJD3	Pro-cancer	Gastric cancer	High-level expression of JMJD3 mediates tumor staging and poor prognosis	[132]
		Cervix cancer	High-level expression of JMJD3 induces epithelial mesenchymal transformation	[133]
		Non-small cell lung cancer	High-level expression of JMJD3 induces tumor cell invasion and metastasis	[134]
		Glioblastoma	High-level expression of JMJD3 and CXCL12 induces tumor cell invasion and distant metastasis	[135]
		Osteosarcoma	High-level expression of JMJD3 mediates cisplatin resistance in tumor cells	[136]
		Prostate cancer	High-level expression of JMJD3 mediates radiation resistance of tumor cells to radiation therapy	[137]
	Anti-cancer	Breast cancer	JMJD3 overexpression inhibits epithelial mesenchymal transformation and distant metastasis of tumor cells mediated by the Wnt/β-catenin signaling pathway	[138]
			High-level expression of JMJD3 inhibits Oct4 expression and breast cancer stem cell-like features	[139]

Recent studies have increasingly highlighted EZH2 as a key driver of tumor progression. Elevated EZH2 expression has been observed in various cancers, including breast, prostate, lung, and thyroid, and is linked to poor prognosis. The retinoblastoma protein (pRB)-E2F transcription factor (E2F) pathway, crucial for regulating the mammalian cell cycle and gene transcription, is often dysregulated in cancer. EZH2 and EED act as downstream regulators of this pathway, with phosphorylated E2F binding to the EZH2 and EED promoters, disrupting the tumor cell cycle and driving malignant progression [140]. For example, EZH2 expression is higher in breast cancer lymph node metastases compared to primary tumor tissues, suggesting that it may be a downstream epigenetic event of E2F activation [118]. Significant reduction in EZH2 mRNA levels was observed in ovarian cancer stem cells transfected with miRNA-98, which was associated with cell cycle arrest and reduced proliferation rates. Co-precipitation and protein blotting analyses showed inhibition of the pRb-E2F signaling pathway [119]. Under hypoxic conditions in the tumor microenvironment, hypoxia-inducible factorα expression was significantly upregulated. EZH2 expression, influenced by hypoxia-inducible factorα binding to hypoxia-responsive elements and promoter regions, plays a key role in tumor cell angiogenesis, metastasis, and invasion. Furthermore, the sodium phosphate transporter solute carrier family 34 member 2 (SLC34A2) is highly expressed in various tumors, where it promotes EZH2 upregulation, contributing to colorectal cancer (CRC) proliferation, metastasis, and treatment resistance in both in vitro and in vivo models. Mechanistically, SLC34A2 activates the EZH2 promoter, indirectly enhancing transcriptional activity through reactive oxygen species accumulation, which stabilizes hypoxia inducible factor 1 alpha [120]. Additionally, the upregulation of EZH2, as a downstream target of the MEK/ERK/Elk signaling pathway, correlates with invasion, proliferation, and poor prognosis in triple-negative breast cancer [121].

While EZH2 is widely recognized as a driver of tumor progression and poor prognosis, its role as a tumor suppressor in specific cancer types also warrants attention. Deletion of the EZH2 allele accelerates lung adenocarcinoma progression in a kirsten rat sarcoma viral oncogene homolog (KRAS)-mutant mouse model, with downstream activation of AKT and ERK pathways promoting tumorigenesis [122]. ZH2 deletion also impairs pancreatic injury repair and regeneration, facilitating KRasG12D-mediated pancreatic carcinogenesis [123]. In addition, mutation or inactivation of the EZH2 gene in myelodysplastic syndrome [124] and T-cell acute lymphoblastic leukemia (T-ALL) [125], where its tumor suppressor role is strongly supported.

Although UTX and JMJD3 share highly similar sequences in their catalytic structural domains, they differ in their roles in tumor pathophysiology. UTX, often acting as a tumor suppressor, has garnered significant attention in bladder carcinogenesis and its malignant progression. As one of the most relevant cancer-associated genes in uroepithelial carcinogenesis, UTX mutations lead to the loss of function of the active region of desmethyltransferase and reduced expression of the downstream target insulin-like growth factor binding protein 3, which significantly contributes to tumorigenesis and high recurrence rates in bladder cancer [126]. However, UTX gene deletion alone is not sufficient to induce bladder carcinogenesis. Dysplastic non-muscle invasive bladder cancer requires a combination of UTX gene defects and p53 haploinsufficiency. UTX gene defects increase the expression of inflammation-related cytokine genes and promote inflammatory cell aggregation, contributing to bladder carcinogenesis by activating inflammatory signaling pathways and increasing the proportion of cancer stem cells [141]. Additionally, The tumor-suppressive role of UTX has been demonstrated in pancreatic cancer, where in vivo and in vitro studies show that enforced UTX expression inhibits pancreatic cancer cell proliferation and invasion, while UTX knockdown is associated with poor prognosis. Mechanistically, low UTX expression restricts GATA binding protein 6-mediated epithelial-mesenchymal transition and typical differentiation, thereby effectively suppressing malignant progression in pancreatic cancer [127]. Immunohistochemical analyses have also shown that downregulation of UTX is associated with poor prognosis in clear cell renal cell carcinoma. Interestingly, both UTX and EZH2 are upregulated in renal cell carcinoma, with the upregulation of UTX expression potentially acting as a compensatory mechanism to counteract fluctuations in H3K27 methylation. Alternatively, this could reflect tumor cell heterogeneity, where UTX and EZH2 serve distinct roles in different tumor cell populations [128].

Despite the established tumor-suppressive function of UTX, some experimental results suggest its potential role as a tumor promoter. For instance, mouse xenograft assays indicate that UTX may promote the proliferation and invasion of breast cancer cells. In this context, UTX and mixed-lineage leukemia 4, which catalyzes H3K4 methylation, work synergistically to regulate gene transcription involved in breast cancer progression, correlating with poor clinical prognosis. This highlights the complex interplay of epigenetic modifiers in tumor biology [129]. Furthermore, UTX acts as a downstream target of the estrogen receptor, enhancing estrogen receptor-positive breast cancer tumorigenesis by establishing a more favorable chromatin state. This interaction activates the expression of C-X-C chemokine receptor type 4, a key driver of metastasis and progression in estrogen receptor-positive breast cancer [130]. UTX mutations have also been observed in male T-ALL patients, and in vitro studies suggest that UTX plays a role as an epigenetic regulator in T-ALL suppression [142]. In T-ALL characterized by T-cell acute lymphocytic leukemia 1 expression, UTX promotes the expression of TAL1-targeted genes, thus maintaining disease progression [131].

Similar to EZH2, JMJD3 also exhibits a dual role in tumorigenesis, with its tumor-promoting or suppressive effects influenced by the environmental and genetic characteristics of tumor cells. In gastric cancer, JMJD3 levels are significantly elevated compared to normal tissues and correlate with tumor stage and poor prognosis [132]. Elevated JMJD3 and UTX expression are also observed in cervical cancer, where inhibiting JMJD3 leads to an increase in H3K27me3 deposition in transcription initiation regions, effectively suppressing epithelial-mesenchymal transition [133]. In non-small cell lung cancer, high JMJD3 levels are associated with high metastatic and invasive rates, and transforming growth factor-β cytokines further activate tumor metastasis [134]. The desmethyltransferase activity of JMJD3 also activates C-X-C motif chemokine ligand (CXCL) 12 expression, which promotes glioma cell invasion and metastasis [135]. JMJD3 function is regulated by the cytokine-responsive transcription factor STAT3, and inhibition of JMJD3 is necessary for maintaining the self-renewal of glioblastoma stem cells [143]. In osteosarcoma, elevated JMJD3 levels correlate with cisplatin resistance, and inhibiting JMJD3 restores tumor sensitivity to cisplatin by inactivating the RAF/ERK/MAPK pathway and silencing protein kinase C alpha and myeloid cell leukemia 1 [136]. In prostate cancer, biopsies from radiation therapy-treated patients show elevated p53 and JMJD3 expression, suggesting that JMJD3 contributes to radiation resistance. JMJD3 demethylase activity activates p53 in response to DNA damage induced by ionizing radiation. Studies indicate that JMJD3 inhibitors may overcome radioresistance in tumor models that otherwise do not respond to radiation therapy [137].

JMJD3 inhibitors also inhibit tumor cell proliferation and metastasis in xenograft models of human breast cancer. Overexpression of JMJD3 can suppress epithelial-mesenchymal transition and metastasis to the lungs through the Wingless-related integration site (Wnt)/β-catenin pathway, further supporting its role as a tumor suppressor [138]. Octamer-binding transcription factor 4, a key factor in maintaining stem cell properties in tumor cells, can be reversed by JMJD3 in breast cancer, and the vitamin D receptor agonist paricalcitol has been shown to modulate breast cancer malignancy by activating the inhibitory effects of JMJD3 on octamer-binding transcription factor 4 [139]. Furthermore, vitamin D has been shown to induce JMJD3 expression in other cancers, and the correlation between 1,25(OH)2D3, vitamin D receptors, and JMJD3 expression may play a crucial role in delaying CRC progression [144].

**Fig. 3:**
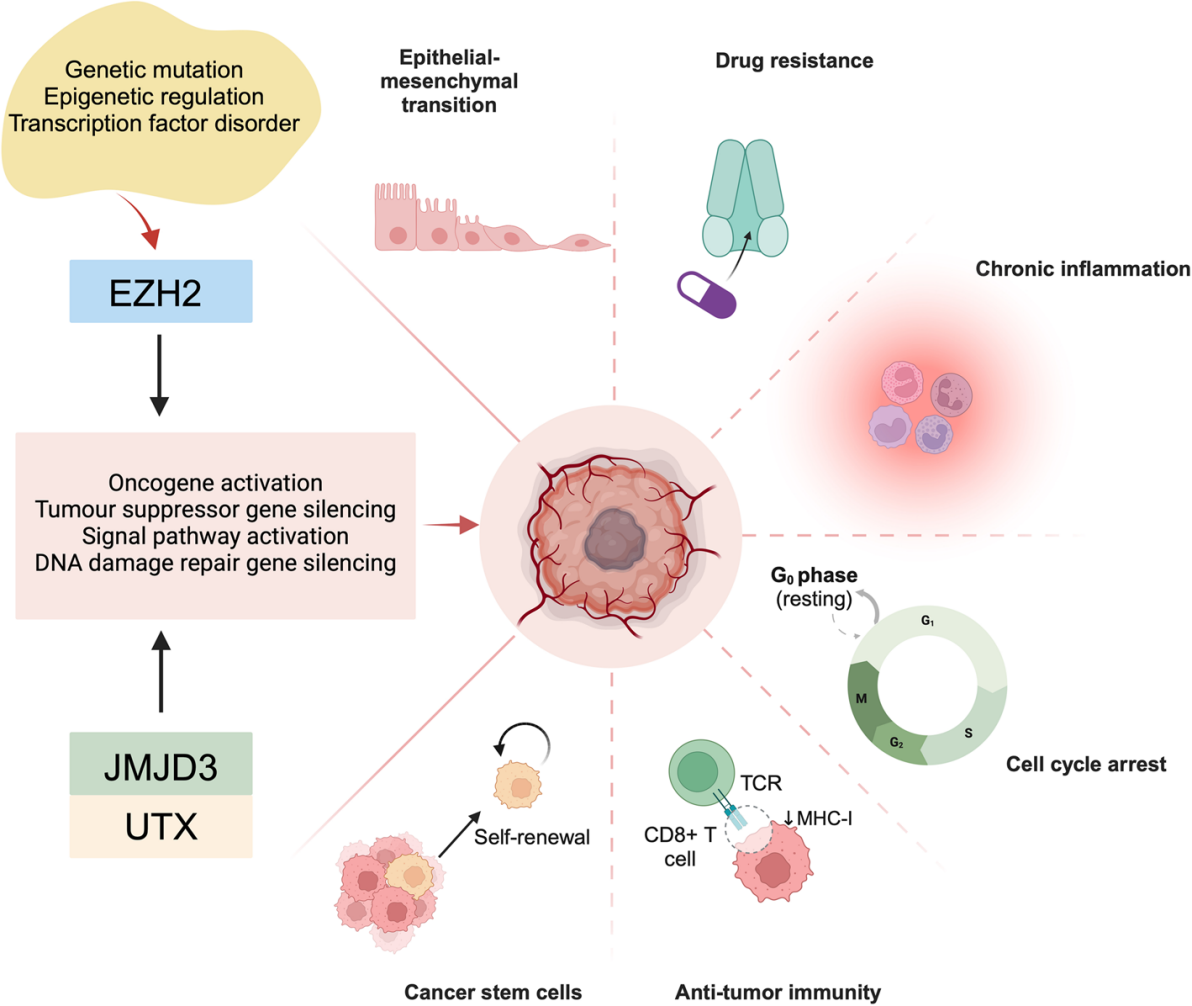
Role of epigenetic modifying enzymes involved in H3K27 methylation modification-related epigenetic modifications in tumorigenesis. EZH2, JMJD3 and UTX often exhibit aberrant expression levels in tumorigenesis, a process that is regulated by mutations, epigenetic modifications and transcription factors. H3K27 methylation modifications mediated by EZH2, JMJD3 and UTX induces the development of a variety of solid and hematological tumors and mediates a variety of biological behaviors, including immune escape, cell-cycle blockade, cancer stem cell self-renewal and drug resistance. Figure was created in https://BioRender.com

In conclusion, the regulation of malignant tumor behaviors through H3K27 methylation has become an important area of study, as both histone methyltransferases and desmethyltransferases play dual roles in gene expression, yielding differential pathological significance and prognostic outcomes across various cancers. The transfer of methyl modifications on chromatin states produces complex biological effects, and further exploration of the biological importance of H3K27 methylation and its modifiers will remain a critical research focus (Fig. 3).

### Interaction of H3K27 methylation with the tumor microenvironment

Tumor cells, immune cells, and stromal cells interact with components like lymphatics and blood vessels to form a dynamic and complex tumor microenvironment (TME). Expanding the focus from tumor cytogenetic and epigenetic events to the interactions within the TME provides a more comprehensive understanding of tumor cell plasticity under immune selective pressure, and how tumor cell phenotypes conducive to survival in a suppressive immune state develop [145]. Among these interactions, H3K27 methylation not only directly affects tumor cell behavior but also regulates the TME, often promoting immune suppression and immune escape [146, 147].

Myeloid-derived suppressor cells (MDSCs), immunosuppressive cells activated during tumor progression, can inhibit specific immune responses and contribute to tumor resistance to immunotherapy [148]. EZH2, which maintains hematopoietic progenitor cell function through chromatin methylation, has been shown ineffective in immunocompetent mice as it is in immunodeficient mice. GSK126, an EZH2 inhibitor, induced MDSC production and suppressed T-cell-mediated tumor immunity, masking its tumor-suppressing effects [149]. However, GSK126 inhibited inflammation in a dextran sodium sulfate -induced colitis model, restoring intestinal homeostasis and slowing colon cancer progression [150]. Given MDSC-mediated tumor immunosuppression, clinical use of EZH2 inhibitors must be approached with caution.

Tumor-associated macrophages (TAMs), particularly the M2-type, are abundant in tumor sites and support immune escape and tumor cell invasion by secreting cytokines and chemokines [151]. Protein arginine methyltransferase facilitates breast cancer metastasis through EZH2 post-translational modifications [152]. In malignant pleural mesothelioma, EZH2 inhibition activates monocyte differentiation into the TAM phenotype, further promoting mesenchymal stem cell growth and extracellular matrix remodeling, which contributes to high invasion rates and poor survival [152]. While EZH2 inhibitors show promise in malignant pleural mesothelioma treatment, their effectiveness is limited by MSC-mediated oncogene activation, suggesting that targeting monocyte recruitment and depleting TAMs may restore the antitumor activity of EZH2 inhibitors [153].

Cytotoxic T lymphocytes directly kill tumor cells by releasing perforin and granzymes, making them key effectors in immune defense. For Cytotoxic T lymphocytes to function, they must recognize tumor antigens presented on major histocompatibility complex (MHC) class I molecules. PRC2-mediated H3K27 methylation affects the MHC-I antigen processing pathway, potentially triggering immune escape by repressing antigen presentation. The reversible epigenetic regulation by PRC2 suggests that combining chromatin complex inhibitors with immunotherapy could overcome tumor progression associated with defective MHC-I molecules [154].

CD4^+^ T cells, upon activation, differentiate into various subpopulations that play distinct roles in antitumor immune responses based on cytokine secretion. However, regulatory T cells (Tregs) derived from CD4^+^ T cells suppress effector T cell-mediated immune defense, maintaining peripheral tolerance and regulating autoimmune responses. Transcription factors are critical in T cell differentiation, and epigenetic signaling influences the plasticity and phenotype of helper and regulatory T cells [155]. JMJD3 and UTX are essential for maintaining H3K27 methylation levels and proper T cell differentiation [156]. In ovarian cancer models, EZH2-mediated histone methylation and DNA methyltransferase programmed death-ligand 1(PD-L1)-mediated DNA methylation inhibit chemokine CXCL9 and CXCL10 expression, promoting tumor aggressiveness. Conversely, combining EZH2 and DNA methyltransferase inhibitors with PD-L1 blockade therapy enhances immune cell infiltration and suppresses tumor growth [157].

Stimulated by inflammatory signals, fibroblasts in tissues are transformed into cancer-associated fibroblasts (CAFs), and the corresponding changes in the local microenvironment of the tumor produce cytokines and chemokines, which provide advantages for the proliferation and survival of tumor cells. In general, high levels of CAFs are associated with malignant biological behavior and poor prognosis of tumors [158]. Considering that CAFs are not composed of a single cell, the tumor suppressive effects exerted by cells with different effects have also been consistently demonstrated experimentally [159]. The H3K27me3 pattern of CAFs is noteworthy, as reduced H3K27me3 levels are usually accompanied by down-regulation of the expression levels of genes that maintain the stemness of cancer cells, and greater fluctuations in H3K27me3 are indicative of more aggressive and invasive tumor cells [160]. In response to inflammatory factor stimulation, downregulation of EZH2 confers a lower level of H3K27 methylation pattern in senescent CAFs for maintenance of senescence-associated secretory phenomena, and the subsequent activation of JAK/STAT3 signaling induces metastasis of cancer cells to the peritoneum and suggests poor prognosis [161].

**Fig. 4:**
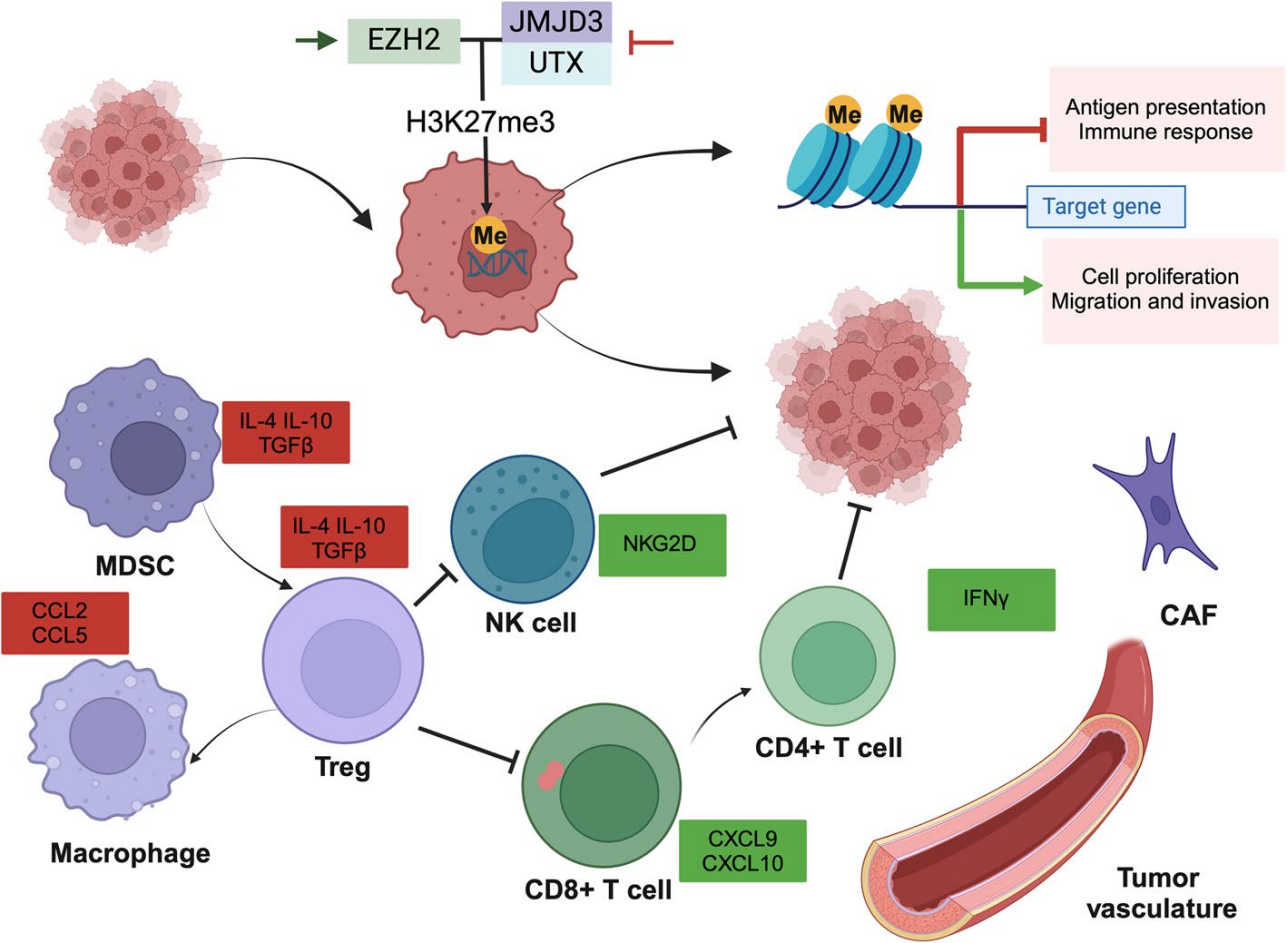
Role of H3K27 methylation in the tumor microenvironment. H3K27 methylation plays a regulatory role for the TME in which tumors survive while affecting their biological behavior. Overall, H3K27me3 forms and maintains the tumor immunosuppressive microenvironment by regulating immune cells to influence the secretion and expression of antigen presentation and chemokines, which help tumor cells evade immune surveillance and inhibit the occurrence of specific immune responses. The red squares represent chemokines, cytokines and receptors with elevated expression, and the green squares represent chemokines, cytokines and receptors with reduced expression. Figure was created in https://BioRender.com

The H3K27 methylation landscape in TAMs highlights a dual biological and immunological mechanism through epigenetic modifications, with selective epigenetic programs shaping the function of infiltrating immune cells and influencing tumor-specific immune responses. Addressing the complex and variable TME, the development of epigenetic drugs combined with immune checkpoint inhibitors may offer strategies to activate immune defense responses and curb tumor progression (Fig. 4).

## Polycomb group proteins and LncRNAs regulate H3K27 methylation

### Roles of polycomb group proteins and LncRNAs in inflammation

**Fig. 5:**
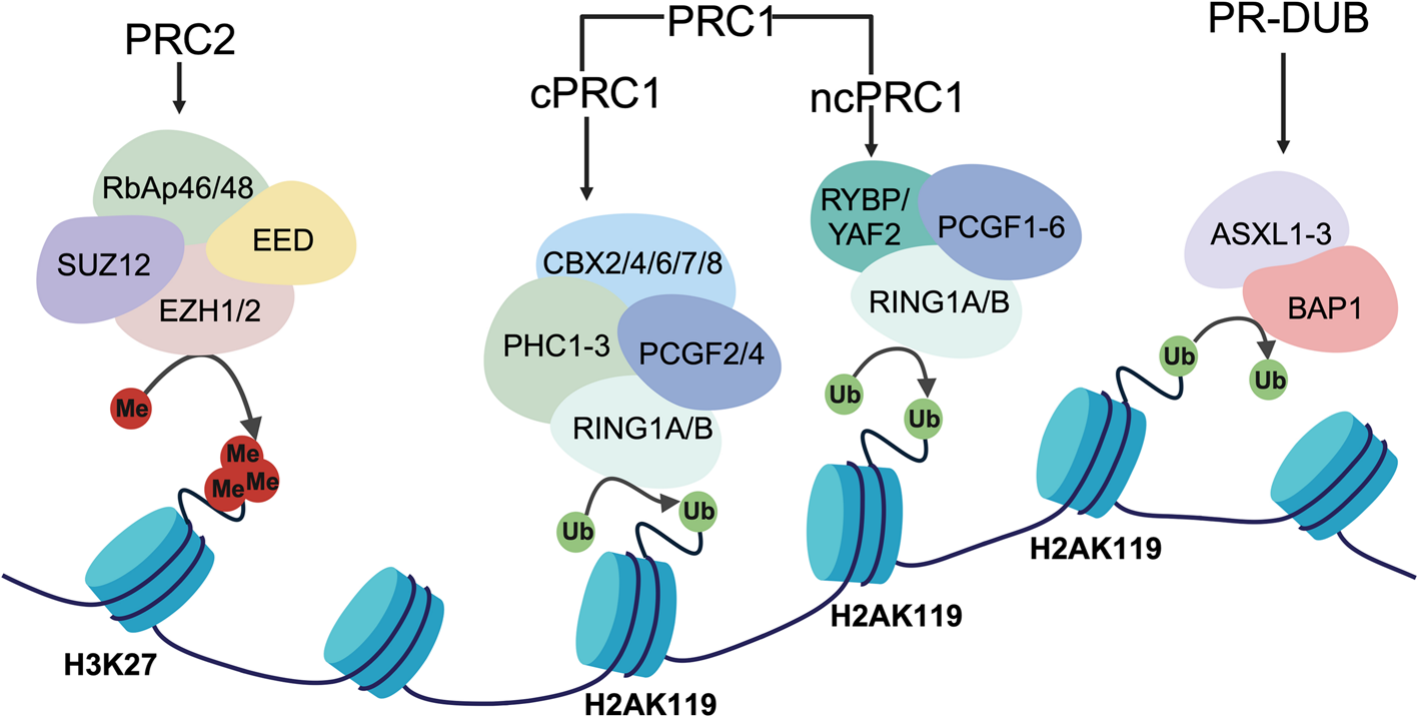
Mammalian PcG complex composition. Mammalian PcG complexes can be divided into three major groups, PRC1, PRC2, and PR-DUB. The PRC2 complex consists of four subunits, EZH1/2, SUZ12, EED, and RbAp46/48. PRC2 is primarily responsible for transferring methyl groups to the histone H3 lysine 27 site and mediating transcriptional repression. PRC1 can be divided into three major groups according to composition and function. cPRC1 and ncPRC1, which are responsible for installing ubiquitination modifications at the histone H2A lysine 119 site. In addition to the shared components RING1A/B and PCGF2/4, cPRC1 has PHC1-3 and CBX2/4/6/7/8. ncPRC1 also has PCGF1/3/5/6 and RYBP/YAF2. PR-DUB, composed of ASXL1-3 and BAP1, is responsible for removing mono-ubiquitylation modifications installed at the histone H2A lysine 119 site chemical modification. Figure was created in https://BioRender.com

PcG proteins are responsible for silencing genes by relying on H3K27me3. In Drosophila, the PcG proteins act as HOX repressors in early developmental stages, and they also exist in mammals as chromatin repressors [162]. A polycomb protein is an essential component of stem cells, progenitor cells, and differentiated cells. It forms three principal multi-protein complexes called PRC1,PRC2 and PR-DUB [163]. The PRC2 protein includes PRC2, EED, SUZ12 polycomb repressive complex 2 subunit, and one of two EZH methyltransferases, which catalyze H3K27me2 and H3K27me3 modification [164]. The PRC1 protein catalyzes the mono-ubiquitination of histone H2A at lysine 119 (H2A119ub1), an epigenetic mark that plays a crucial role in regulating the crosstalk between PRC1 and PRC2 complexes and in repressing gene expression through the PcG pathway [165]. The PRC1 complex, composed of RING1A/B-PCGF heterodimers, can be classified into canonical PRC1 (cPRC1) and non-canonical PRC1 (ncPRC1) based on protein components and functions. cPRC1 is involved in chromatin condensation and the majority of H2A119ub1 production, and includes RING1A/B, polycomb group ring finger (PCGF) (PCGF2/4), polyhomeotic-like (PHC) (PHC1/2/3), and chromobox (CBX) (CBX2/4/6/7/8), while ncPRC1 consists of RING1A/B, PCGF (PCGF1-6), and RYBP/YAF2 [166]. The PR-DUB complex, containing BRCA1 associated protein 1 (BAP1) and additional sex combs-Like proteins (ASXLs) (ASXL1-3), is responsible for removing the H2A119ub1 mark [167]. Increasing evidence indicates that PcG proteins contribute to inflammation and immune evasion in tumors (Fig. 5).

Aberrant histone methylation often accompanies inflammation-driven tumor progression, and studies in mouse intestinal epithelial cells suggest that PcG-mediated transcriptional repression may be linked to such methylation events [168]. In a rat model, downregulation of IEC-6 upon SUZ12 inhibition resulted in upregulation of genes involved in development and immunity, and suppression of IL-1-induced inflammatory responses, highlighting the role of PRC2 in regulating cell growth and inflammation [169]. In KRAS-driven non-small cell lung cancer, EED deficiency leads to IL6 expression and macrophage aggregation, contributing to sterile inflammation and impaired lung tissue function [170]. Accumulating experimental results suggest that the effect of mediating inflammatory responses in PcG proteins is dependent on the dysregulation of the expression of specific components, and therefore attempts to combine epigenetic and anti-inflammatory therapies are a therapeutic modality to be considered. Notably, immune cell-mediated secretion of inflammatory cytokines is a key component in driving the intracellular inflammatory response [171], and existing studies support the idea that PcG proteins have an immunomodulatory role. In metastatic cutaneous squamous cell carcinoma, elevated levels of RING1B and EZH2 inhibit inflammation and promote immune evasion by suppressing the expression of inflammation-related genes and chemokines. In contrast, lower levels of PcG proteins in non-metastatic squamous cells correlate with increased immune response and inflammation, indicating that PcG proteins modulate immune activation and inflammatory reactions [172].

The growing list of evidence regarding the role of long non-coding RNAs (lncRNAs) in recruiting PRC1 or PRC2 as a means of establishing repressive chromatin states provides a basis for interpreting potential three-dimensional interactions between lncRNAs and PcG proteins [173]. In addition to this, the involvement of lncRNAs in the regulation of inflammatory responses by mediating H3K27me3 levels in inflammatory mediator promoters is also worth exploring. The up-regulation of lnc-IL7R expression and the corresponding increase in H3K27me3 levels in the promoter region of inflammatory mediators in response to LPS stimulation suggest that lnc-IL7R can inhibit excessive inflammatory responses by regulating gene silencing mediated by H3K27me3 expression [174]. Antisense transcribed long noncoding RNA antisense non-coding RNA in the INK4 locus (ANRIL) can recruit PcG proteins to exert their inhibitory transcriptional roles and participate in disease by regulating multiple signaling pathways. Inflammation is a driving factor in the development of atherosclerotic vascular disease, and the role played by ANRIL in the pathogenesis of atherosclerotic vascular disease is dependent on different shear forms [175]. Sepsis is a severe life-threatening clinical syndrome characterized by immune cell death and increased inflammatory response. lncRNA urothelial cancer associated 1 has been shown to bind EZH2 to inhibit the inflammatory response in which homeobox A1 is involved in the induction of septic pneumonia [176]. Another lncRNA metastasis-associated lung adenocarcinoma transcript 1 can downregulate ubiquitin specific peptidase 22 expression levels by binding to EZH2, which induces an inflammatory response leading to myocardial injury and thus promotes septic myocardial dysfunction [177].

### The role of polyribonucleic acid histones and LncRNAs in cancer

In mammalian cancer progression, aberrant epigenetic regulatory networks, often mediated by PcG protein dysregulation, frequently play dual roles in tumor development-both oncogenic and oncostatic. In double-negative prostate cancer, the pro-carcinogenic function of PRC1 is mediated through activation of downstream target chemokine (C–C motif) ligand 2. This process helps maintain stem cell-like properties of tumor cells, while also recruiting immune cells such as TAMs and Tregs. These immune cells facilitate immune evasion and create a tumor microenvironment that supports metastasis [178]. High PRC1 expression confers tumor cell resistance to immune surveillance and enhances self-renewal capacity, promoting the proliferation and metastasis of hepatocellular carcinoma [179]. Recent experimental results suggest that depletion of PcG proteins drives tumor transformation [179]. Experimental data suggests that depletion of PcG proteins can drive tumor transformation, underscoring their critical role in cancer development. For example, in the context of the KRAS G12D mutation, deletion of chromobox 4 destabilizes chromosomes and activates multiple signaling pathways, including the Hippo pathway, which are essential for lung adenocarcinoma progression [180]. Similarly, in hepatocellular carcinoma, upregulation of CBX4 expression stimulates angiogenesis and worsens prognosis, in part by enhancing hypoxia inducible factor 1 alpha transcription to meet the increased oxygen demands of tumor cells [181]. These findings suggest that the roles of PcG proteins in carcinogenesis are context-dependent, influenced by the genetic background and cellular environment of tumor. Consequently, targeting key regulators of chromosomal instability could offer promising therapeutic avenues for cancers associated with aberrant PcG protein function.

Recent research has highlighted the involvement of lncRNAs in PcG-mediated tumor progression. For instance, the mechanism by which lncRNA small nucleolar RNA host gene 22 (SNHG22) promotes gastric cancer progression has been elucidated. Here, the ETS like transcription factor 4 facilitates the interaction between SNHG22 and EZH2, promoting tumor cell survival and proliferation by upregulating NOTCH1 expression [182]. Similarly, nuclear paraspeckle assembly transcript 1, another lncRNA, is upregulated in gastric cancer, where it is modulated by the m6A methylation enzyme ALKBH5. This interaction reduces m6A modification and EZH2 expression, suggesting a synergistic role of various epigenetic modifications in cancer development [183]. LncRNA HOX transcript antisense intergenic RNA (HOTAIR) has been implicated in the pathogenesis of many cancers, and developing small-molecule inhibitors targeting HOTAIR remains a key clinical challenge. One such drug, AC1Q3QWB (AQB), targets the interaction between HOTAIR and EZH2, inhibiting PRC2-mediated epigenetic repression. AQB has shown therapeutic promise in endometrial cancer by suppressing tumor cell proliferation and the cell cycle, especially when combined with the EZH2 inhibitor tazemetostat [184]. These findings suggest that lncRNA-targeting agents, in combination with EZH2 inhibitors, may offer a novel and effective approach for cancers linked to abnormal PcG function.

## Drug modulation H3K27 methylation

The preeminent role of H3K27 methylation status and enzymes related to the regulation of methylation status in inflammatory responses and cancer progression is continuously validated, and the search for more potent epimedic agents and their introduction into preclinical and clinical studies is significant at this stage. Here, we will enumerate the evidence for small molecule inhibitors targeting key enzymes that regulate H3K27me3 in modulating inflammation and driving cancer.

Small molecule inhibitors of EZH2, such as DZNep, have demonstrated promising anticancer effects by accumulating SAH and preventing the transfer of methyl groups from SAM to histone residues. DZNep has been shown to inhibit the proliferation of gastric cancer cells in a dose- and time-dependent manner, partly through suppression of hypoxia-inducible factor 1α and Wnt/β-catenin signaling pathways [185]. Furthermore, DZNep has demonstrated efficacy in reducing inflammatory cell infiltration and symptoms in allergic airway inflammation [186]. In addition to its effects on H3K27me3, DZNep can also impact other histone methylation marks such as H4K20me3 and H3K4me3 [187]. Based on high-throughput screening, an increasing number of SAM-competitive inhibitors of EZH2 are being discovered and utilized. Tazemetostat, an oral EZH2 inhibitor, is approved for treating epithelioid sarcoma, and is under investigation for other cancers [188]. Tazemetostat can modulate immune responses and inhibit tumor progression. For example, in co-cultures of macrophages and CRC cells, Tazemetostat promotes macrophage polarization to a pro-inflammatory phenotype, thereby enhancing anti-tumor immunity [189]. GSK126, another EZH2 inhibitor, is able to exert an advantage in inhibiting proliferation and blocking the cell cycle in lymphomas and solid tumors, either alone or in combination with other drugs [190, 191], and inhibit angiogenesis by downregulating vascular endothelial growth factor A expression [192]. GSK126 is effective in inhibiting CRC cell invasion in combination with an farnesoid X receptor (FXR) agonist (obeticholic acid) in a virtually side-effect-free manner, a process that is dependent on activation of caudal type homeobox 2 expression and accelerated nuclear localization of FXR. However, GSK126 did not exert the expected efficacy in a phase I clinical trial [193]. GSK126, when combined with FXR agonists like obeticholic acid, can inhibit CRC cell invasion with minimal side effects, activating CDX2 expression and promoting nuclear localization of FXR. However, its efficacy was limited in phase I clinical trials due to the induction of MDSCs, which suppressed T-cell-mediated anti-tumor immunity [149]. Future studies could focus on overcoming GSK126 toxicity and attempting to combine it with other therapies to explore the best way for GSK126 to fulfill its antitumor therapeutic potential.

EZH1 and EZH2 are highly homologous proteins that both contribute to the maintenance of H3K27 methylation. In hepatocellular carcinoma, combined knockdown of EZH1 and EZH2 has shown greater tumor suppression than targeting EZH2 alone, suggesting that dual inhibitors could be more effective in certain cancers [194]. Sorafenib, a first-line therapeutic agent for the treatment of hepatocellular carcinoma, increases H3K27me3 levels in tumor cells and induces EZH2 activation after long-term treatment. When Sorafenib was combined with UNC1999, a dual EZH1/2 inhibitor, UNC1999 was able to reverse the increased H3K27me3 levels and synergize with Sorafenib to exert stronger antitumor effects [194]. In addition In addition, UNC1999 has been shown to reduce bladder cancer proliferation by targeting the JAK2/STAT3 signaling pathway [195]. In addition, UNC1999 can reactivate the sensitivity of bladder cancer tumor cells to radiation therapy by decreasing the expression of EZH2 [196]. A phase I/II clinical trial with the dual inhibitor valemetostat (DS-3201b) in combination with irinotecan for small cell lung cancer was terminated early due to exceeding toxicity thresholds. However, it demonstrated some efficacy, indicating that combining valemetostat with other agents that do not induce excessive toxicity may provide a promising treatment strategy for small cell lung cancer [197].

Blocking the JMJ subfamily decreases the production of proinflammatory cytokines in human primary macrophages stimulated by LPS, a process that relies on JMJD3 and UTX. This indicates that H3K27-specific JMJs play a role in regulating disease-related inflammatory responses [198]. GSK-J4, a specific blocker of JMJD3 and UTX, reduces the release of inflammatory cytokines IL-6, interferon gamma, and TNF [199]. GSK-J4 could inhibit intestinal injury and inflammation by regulating NF-κB and JAK2/STAT3 pathway [200]. In addition, GSK-J4 has been shown to play a role in maintaining the tolerance phenotype of DCs and in effectively slowing the inflammatory response in autoimmune encephalomyelitis, highlighting its potential for treating autoimmune diseases [199]. Notably, the potent demethylase inhibition of GSK-J4 targets multiple signaling pathways to exert anticancer effects across various cancer types, demonstrating promising synergistic anticancer properties when combined with other therapies. Experimental results have shown that the combination of GSK-J4 and donafenib activates ferroptosis and effectively kills hepatocellular carcinoma cells [201]. Similarly, a combination of low-dose GSK-J4 and cabazitaxel induces cell death in castration-resistant prostate cancer tumor cells [202]. Importantly, GSK-J4 is a broad-spectrum demethylase inhibitor, targeting UTX, JMJD3, and other demethylases with Jmjc structural domains [203]. Furthermore, a computerized screening identified another JMJD3 inhibitor, the benzoxazole derivative 8, which operates in the low micromolar range and shows potential for melanoma therapy [204]. In 2020, Zhang et al. developed a capillary electrophoresis technique and successfully screened two JMJD3 inhibitors, salvianic acid A and puerarin 6′'-O-xyloside [205] (Table 3).

**Table 3:** Inhibitors of EZH2, JMJD3 and UTX and their mechanisms of action

Target	Drug	Mechanism	Phrase	Refs
EZH2	DZNep	SAH hydrolase inhibitor of methyltransferases	Preclinical	[185]
	Tazemetostat (EPZ6438)	SAM-competitive inhibitor of PRC2	Phase 1/2	[188]
	GSK126	SAM-competitive inhibitor of PRC2	Phase 1	[190, 191]
EZH1/2	UNC1999	SAM-competitive inhibitor of PRC2	Preclinical	[194]
	Valemetostat (DS-3201B)	SAM-competitive inhibitor of PRC2	Phase 1	[197]
JMJD3/UTX	GSK-J4	The ethyl ester derivative of GSK-J1	Preclinical	[198]
JMJD3	Benzoxazole derivative 8	A fragment-based approach and the computer-aided strategy	Preclinical	[204]
	Salvianic acid A and Puerarin 6′’-O-xyloside	Identified by capillary electrophoresis	Preclinical	[205]

In summary, methyltransferases and demethyltransferases that regulate H3K27 methylation play crucial roles in inflammatory responses, immune cell function, and carcinogenesis. The development of small molecule inhibitors targeting H3K27me3 has garnered significant interest. Available findings indicate that drugs targeting H3K27me3 can effectively inhibit tumor progression and overcome resistance to anticancer drugs, either alone or in combination with other therapies.

## Conclusions and future prospects

Inflammation serves as an immune defense mechanism, enabling tissue modification and the elimination of abnormal cells. However, chronically activated inflammation is recognized as a potential catalyst for tumor progression. Cancer progression is intricately regulated by both tumor cells and immune cells. These cells can reshape epigenetic modifications, thereby creating an inflammatory microenvironment and sustaining the inflammatory state through the regulation of the global epigenetic landscape. Given the shared effectors in inflammation and cancer progression, epigenetic modifications are assuming ever—greater significance in deciphering inflammation, cancer, and the interactions between them.

A growing body of research underscores the regulatory importance of H3K27 methylation in key biological processes, including inflammatory responses, carcinogenesis, and immune regulation. The repressive epigenetic effects of H3K27 methylation interact with various signaling pathways to precisely modulate chromatin structure and transcriptional states, maintaining methylation homeostasis. The methyltransferase EZH2 and the demethylases JMJD3 and UTX, responsible for regulating H3K27 methylation, exert complex and context-dependent effects on gene expression, often displaying dual roles in tumorigenesis and progression. This suggests that the expression of these epigenetic enzymes is influenced by both genetic and epigenetic factors, and their functions depend on tumor type and cellular genetic context.

Despite the promise of drugs targeting H3K27 methylation in epigenetic therapy, most of these inhibitors remain in preclinical stages. Current drugs face challenges such as instability and unclear mechanisms of action, which prevent them from meeting the urgent clinical demand for effective epigenetic treatments. As a result, the search for more efficient H3K27 methylation-targeted drugs is critical. With ongoing advancements in the identification of H3K27me3 inhibitors, we anticipate the development of more optimized epigenetic drugs that will enhance the diagnosis and treatment of various diseases.

Given the significant role of H3K27 methylation and the related regulatory enzymes in promoting tumor progression, future breakthroughs are expected in understanding its impact on the tumor microenvironment. Additionally, the roles of PRC1 subunits and EZH1 proteins in inflammation and cancer are not fully understood. Given their essential functions in PRC1 and EZH1, exploring their roles in inflammation and tumor progression could open promising avenues for therapeutic development.

## Data Availability

Not applicable.
